# Prevalence of Self-Reported Intake of Sugar-Sweetened Beverages Among US Adults in 50 States and the District of Columbia, 2010 and 2015

**DOI:** 10.5888/pcd18.200434

**Published:** 2021-04-15

**Authors:** Jennifer R. Chevinsky, Seung Hee Lee, Heidi M. Blanck, Sohyun Park

**Affiliations:** 1 Division of Nutrition, Physical Activity, and Obesity, National Center for Chronic Disease Prevention and Health Promotion, Centers for Disease Control and Prevention, Atlanta, Georgia; 2 Epidemic Intelligence Service, Centers for Disease Control and Prevention, Atlanta, Georgia

## Abstract

Frequent intake of sugar-sweetened beverages (SSBs) is associated with adverse health outcomes, including obesity, type 2 diabetes, and cardiovascular disease. We used combined data from the 2010 and 2015 National Health Interview Survey to examine the prevalence of SSB intake among US adults in all 50 states and the District of Columbia. Approximately two-thirds of adults reported consuming SSBs at least daily, including more than 7 in 10 adults in Hawaii, Arkansas, Wyoming, South Dakota, Connecticut, and South Carolina, with significant differences in sociodemographic characteristics. Efforts to decrease SSB consumption could consider the sociodemographic and geographic differences in SSB intake when designing equitable interventions.

**Summary**

**What is already known about this topic?**

Frequent intake of sugar-sweetened beverages (SSBs) is associated with adverse health consequences. SSB intake differs by geographical region and sociodemographic characteristics.

**What is added by this report?**

We report SSB intake by state for all 50 states and the District of Columbia along with notable geographic and sociodemographic differences.

**What are the implications for public health practice?**

Efforts to decrease SSB intake could consider sociodemographic and geographic differences in SSB intake to inform design of interventions.

## Objective

Sugar-sweetened beverages (SSBs) are a leading source of added sugars in the US diet and are associated with obesity, type 2 diabetes, heart disease, kidney disease, nonalcoholic fatty liver disease, and tooth decay (1–4). SSBs, which are sweetened with various forms of added sugars, include regular soda, sweetened fruit drinks, sports/energy drinks, and sweetened coffee/tea drinks (5). Previous studies reported geographic differences in SSB intake (6–8). However, no study has reported SSB intake for every state. We assessed the prevalence of SSB intake among US adults by sociodemographic characteristics for all 50 states and the District of Columbia by using National Health Interview Survey (NHIS) data.

## Methods

NHIS is a nationally representative, cross-sectional household survey conducted by the National Center for Health Statistics (NCHS) that uses in-person interviews. The Cancer Control Supplement (CCS), which contains dietary intake information, was administered both in 2010 and in 2015 and was approved by the NCHS Research Ethics Review Board. We used nationally weighted data from combined 2010 and 2015 NHIS CCS to examine the prevalence of consuming SSBs 1 or more times daily among 56,260 US adults aged 18 or older. Data were combined to increase the sample size and reduce the variability associated with state estimates. This study required the use of restricted NHIS files for state estimates and categorizing metropolitan status available through the NCHS Research Data Center. SSB intake was based on survey respondents’ answers to 4 questions asking about intake frequency over the past month of regular soda, sweetened fruit drinks, sports/energy drinks, and sweetened coffee/tea drinks (9,10). Sweetened fruit drinks and sweetened coffee/tea drinks included drinks that were presweetened in addition to drinks that were sweetened at home by adding sugar. Adults responded with intake frequency per day, week, or month for each beverage type. Weekly and monthly intake frequency for each type of beverage was converted to daily intake frequency by dividing by 7 or 30, respectively. To calculate frequency of total daily SSB intake, we summed responses from intake of regular soda, sweetened fruit drinks, sports/energy drinks, and sweetened coffee/tea drinks. SSB categories and frequency cutoff of once per day were used, consistent with previous studies (6,7). Differences in respondent characteristics were assessed by χ^2^ tests (*P* < .05). Prevalence estimates were calculated for SSB categories and by state for all 50 states and the District of Columbia. Analyses were conducted with SAS-callable SUDAAN, version 9.0 (RTI) to account for a complex survey design and sampling weights.

## Results

Overall, 63.0% of US adults reported consuming SSBs 1 or more times daily in combined 2010 and 2015 NHIS CCS data (Table 1). US adults reported consuming the following 1 or more times daily, by beverage type: sweetened coffee/tea drinks, 39.5%; regular soda, 19.5%; fruit drinks, 5.7%; and sports/energy drinks, 5.5%. Among sociodemographic categories with significant differences overall, the prevalence of SSB intake was highest among adults aged 18 to 24 (65.0%) and 25 to 39 (65.4%), men (66.1%), Hispanic respondents (70.1%), people with less than a high school education (69.8%), people with an annual household income less than $35,000 (66.0%), people residing in nonmetropolitan areas (65.0%), and people residing in the Northeast census region (67.0%). The prevalence of SSB intake did not significantly differ by marital status. 

**Table 1:** Prevalence of Sugar-Sweetened Beverage Intake Once Daily or More Among US Adults Aged 18 or Older (N = 56,260), National Health Interview Survey Cancer Control Supplement, 2010 and 2015^a^

Characteristic	No. Respondents	≥1 Time/d, Weighted % (95% CI)^b^
**Overall**	56,260	63.0 (62.4–63.6)
**Age, y^b^**		
18–24	5,358	65.0 (63.3–66.7)
25–39	15,027	65.4 (64.4–66.3)
40–59	19,143	62.8 (61.8–63.7)
≥60	16,732	59.7 (58.6–60.8)
**Sex^b^**		
Male	25,148	66.1 (65.3–67.0)
Female	31,112	60.0 (59.3–60.8)
**Race/ethnicity^b^**		
White, non-Hispanic	33,488	61.4 (60.7–62.2)
Black, non-Hispanic	8,238	64.3 (63.0–65.7)
Hispanic	9,984	70.1 (68.7–71.4)
Other, non-Hispanic	4,550	60.5 (58.5–62.5)
**Marital status**		
Married/domestic partnership	28,079	62.7 (61.9–63.4)
Not married	28,181	63.5 (62.7–64.3)
**Education^b^**		
<High school	8,712	69.8 (68.5–71.0)
High school/GED	14,358	67.3 (66.2–68.3)
Some college	17,200	62.8 (61.8–63.8)
College graduate	15,990	56.4 (55.4–57.4)
**Annual household income, $^b^**		
<35,000	23,665	66.0 (65.2–66.9)
35,000–74,999	17,061	64.3 (63.3–65.3)
75,000–99,999	5,744	61.8 (60.1–63.4)
≥100,000	9,790	57.7 (56.4–59.0)
**Metropolitan/nonmetropolitan status^b^^,^ c**		
Metropolitan	46,623	62.7 (62.0–63.3)
Nonmetropolitan	9,637	65.0 (63.2–66.7)
**Census region^b^^,^ d**		
Northeast	9,084	67.0 (65.5–68.4)
Midwest	12,100	58.3 (57.0–59.7)
South	20,072	65.2 (64.2–66.1)
West	15,004	61.1 (59.9–62.2)

a Data are for 50 states and the District of Columbia. The type of SSBs consumed was based on survey respondents’ answers to 4 questions: 1) “During the past month, how often did you drink regular soda or pop that contains sugar? Do not include diet soda.”; 2) “During the past month, how often did you drink sweetened fruit drinks, such as Kool-Aid, cranberry, and lemonade? Include fruit drinks you made at home and added sugar to.”; 3) “During the past month, how often did you drink sports and energy drinks such as Gatorade, Red Bull, and vitamin water?”; and 4) “During the past month, how often did you drink coffee or tea that had sugar or honey added to it? Include coffee and tea you sweetened yourself and presweetened tea and coffee drinks such as Arizona Iced Tea and Frappuccino. Do not include artificially sweetened coffee or diet tea.”

b Significant difference in the prevalence of SSB intake once daily or more across levels of the characteristic at the *P* < .05 level based on χ^2^ test.

c Based on National Center for Health Statistics Urban–Rural Classification Scheme for Counties (https://www.cdc.gov/nchs/data_access/urban_rural.htm). Metropolitan includes large central metro, large fringe metro, medium metro, and small metro categories. Nonmetropolitan includes micropolitan and noncore categories.

d US Census Bureau–defined regions: Northeast (Connecticut, Maine, Massachusetts, New Hampshire, New Jersey, New York, Pennsylvania, Rhode Island, Vermont); Midwest (Illinois, Indiana, Iowa, Kansas, Michigan, Minnesota, Missouri, Nebraska, North Dakota, Ohio, South Dakota, Wisconsin); Southern (Alabama, Arkansas; Delaware, District of Columbia, Florida, Georgia, Kentucky, Louisiana, Maryland, Mississippi, North Carolina, Oklahoma, South Carolina, Tennessee, Texas, Virginia, West Virginia); and Western (Alaska, Arizona, Colorado, Hawaii, Idaho, Montana, Nevada, New Mexico, Oregon, Utah, Washington, Wyoming).

By state, SSB intake of 1 or more times daily ranged from 44.5% in Alaska to 76.4% in Hawaii. These 6 states had a prevalence of daily SSB intake of 70.0% or more: Hawaii (76.4%), Arkansas (74.2%), Wyoming (73.2%), South Dakota (72.5%), Connecticut (72.2%), and South Carolina (70.2%). Only 1 state, Alaska (44.5%), had a daily intake prevalence below 50.0% (Table 2). Most states had a daily intake prevalence between 50.0% and 70.0% (Figure).

**Table 2:** Prevalence by State of Sugar-Sweetened Beverage Intake Once Daily or More Among US Adults Aged 18 or Older, National Health Interview Survey Cancer Control Supplement, 2010 and 2015

State	No. Respondents	Weighted % (95% CI)^a^
Nation overall	56,260	63.0 (62.4–63.6)
Alabama	813	65.0 (60.2–69.6)
Alaska	469	44.5 (40.3–48.8)
Arizona	898	64.5 (59.6–69.1)
Arkansas	602	74.2 (70.2–77.8)
California	6,628	62.7 (61.0–64.3)
Colorado	882	59.4 (55.0–63.6)
Connecticut	652	72.2 (67.8–76.3)
Delaware	463	68.0 (60.5–74.6)
District of Columbia	563	64.8 (57.5–71.4)
Florida	3,184	67.2 (65.2–69.2)
Georgia	1,548	68.1 (65.1–70.9)
Hawaii	516	76.4 (73.9–78.7)
Idaho	531	58.8 (55.0–62.5)
Illinois	1,946	62.7 (59.5–65.8)
Indiana	1,034	65.7 (61.0–70.2)
Iowa	752	50.5 (44.3–56.7)
Kansas	815	54.9 (51.5–58.3)
Kentucky	893	67.2 (62.0–72.0)
Louisiana	787	68.7 (65.2–71.9)
Maine	638	65.5 (63.6–67.3)
Maryland	830	65.4 (61.3–69.3)
Massachusetts	858	66.8 (62.7–70.7)
Michigan	1,437	59.0 (55.1–62.8)
Minnesota	985	50.4 (46.2–54.7)
Mississippi	674	64.5 (61.8–67.0)
Missouri	871	59.1 (55.4–62.7)
Montana	467	64.9 (63.4–66.3)
Nebraska	614	58.0 (54.6–61.3)
Nevada	760	63.8 (58.4–68.8)
New Hampshire	526	69.7 (66.9–72.3)
New Jersey	1,220	69.5 (65.6–73.2)
New Mexico	728	68.5 (65.8–71.1)
New York	2,701	65.6 (63.1–68.1)
North Carolina	1,511	62.7 (59.0–66.2)
North Dakota	506	59.2 (53.8–64.5)
Ohio	1,716	57.2 (54.1–60.3)
Oklahoma	669	66.0 (59.1–72.3)
Oregon	708	51.5 (48.6–54.4)
Pennsylvania	1,727	65.9 (62.6–69.0)
Rhode Island	390	65.7 (58.1–72.6)
South Carolina	739	70.2 (64.6–75.4)
South Dakota	515	72.5 (69.0–75.7)
Tennessee	909	66.4 (61.2–71.2)
Texas	4,227	62.5 (60.3–64.6)
Utah	734	53.6 (49.1–58.1)
Vermont	372	67.3 (64.6–69.8)
Virginia	1,097	59.6 (56.1–63.0)
Washington	1,185	55.0 (51.9–58.0)
West Virginia	563	59.4 (55.8–62.9)
Wisconsin	909	50.4 (46.6–54.2)
Wyoming	498	73.2 (67.7–78.0)

a The type of SSBs consumed was based on survey respondents’ answers to 4 questions: 1) “During the past month, how often did you drink regular soda or pop that contains sugar? Do not include diet soda.”; 2) “During the past month, how often did you drink sweetened fruit drinks, such as Kool-Aid, cranberry, and lemonade? Include fruit drinks you made at home and added sugar to.”; 3) “During the past month, how often did you drink sports and energy drinks such as Gatorade, Red Bull, and vitamin water?”; and 4) “During the past month, how often did you drink coffee or tea that had sugar or honey added to it? Include coffee and tea you sweetened yourself and presweetened tea and coffee drinks such as Arizona Iced Tea and Frappuccino. Do not include artificially sweetened coffee or diet tea.”

**Figure:**
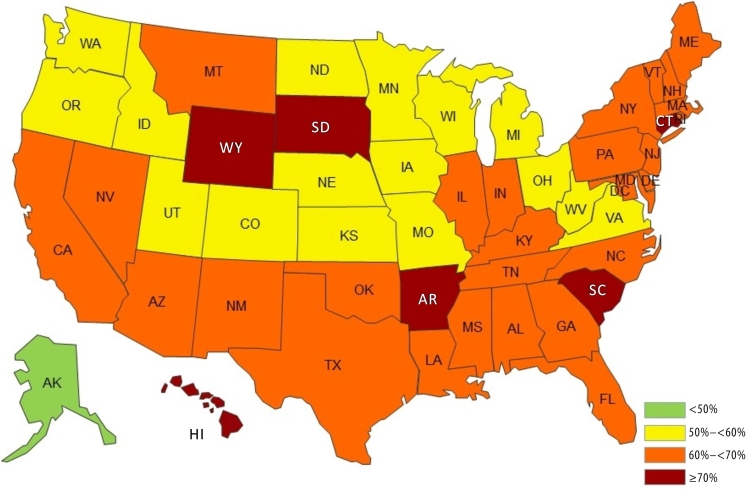
Prevalence of self-reported sugar-sweetened beverage (SSB) intake once daily or more among US adults by state, National Health Interview Survey Cancer Control Supplement (NHIS CCS), 2010 and 2015. SSBs include regular soda, sweetened fruit drinks, sports/energy drinks, and sweetened coffee/tea drinks. This map shows combined 2010 and 2015 data from the NHIS CCS (9,10).

**Table:** 

State	Prevalence %
Alabama	65.0
Alaska	44.5
Arizona	64.5
Arkansas	74.2
California	62.7
Colorado	59.4
Connecticut	72.2
Delaware	68.0
District of Columbia	64.8
Florida	67.2
Georgia	68.1
Hawaii	76.4
Idaho	58.8
Illinois	62.7
Indiana	65.7
Iowa	50.5
Kansas	54.9
Kentucky	67.2
Louisiana	68.7
Maine	65.5
Maryland	65.4
Massachusetts	66.8
Michigan	59.0
Minnesota	50.4
Mississippi	64.5
Missouri	59.1
Montana	64.9
Nebraska	58.0
Nevada	63.8
New Hampshire	69.7
New Jersey	69.5
New Mexico	68.5
New York	65.6
North Carolina	62.7
North Dakota	59.2
Ohio	57.2
Oklahoma	66.0
Oregon	51.5
Pennsylvania	65.9
Rhode Island	65.7
South Carolina	70.2
South Dakota	72.5
Tennessee	66.4
Texas	62.5
Utah	53.6
Vermont	67.3
Virginia	59.6
Washington	55.0
West Virginia	59.4
Wisconsin	50.4
Wyoming	73.2

## Discussion

Daily SSB intake is common among US adults and is particularly high in some states and among some populations. The prevalence in our study was higher than in the 2017 Behavioral Risk Factor Surveillance System (BRFSS) survey (8). This discrepancy may be explained by differences in the types of SSBs assessed, modes of survey administration, methods of collecting dietary intake data, and representativeness. Previous NHIS, NHANES (National Health and Nutrition Examination Survey), and BRFSS data also showed that SSB consumption is higher among young adults, men, adults in nonmetropolitan counties, and people with low levels of education (6–8,11).

The prevalence of SSB consumption in previous studies was high in the Northeast (7) and in southern states (6), consistent with our study’s findings. The high northeastern prevalence may be due to high consumption of sweetened coffee or tea drinks (7). Data from the 2017 BRFSS survey (8) for 12 states, and data from the 2013 BRFSS survey (6) for 23 states also revealed state-specific differences in SSB intake. Reasons for state differences may reflect demographic differences. States and communities may also differ in SSB marketing (12), pricing, and access to alternatives.

Our study has several limitations, including self-reported information, assessment of intake frequency without volume or amount of SSBs, age of the data, and combination of data. Declines in SSB intake have occurred over time (13). Combining data may mask changes in prevalence in the study period. Regardless, ours is the first study to our knowledge to examine SSB intake frequency for all 50 states and the District of Columbia by using a nationally representative sample of US adults. Our findings highlight that prevalence of daily SSB intake remains high among US adults, with sociodemographic and geographic differences. Efforts to decrease SSB intake could consider the higher intake prevalence in sociodemographic and geographic subpopulations to aid design and targeting of equitable interventions.
